# The Spread of Poliomyelitis in America

**Published:** 1910-10-08

**Authors:** 


### The Hospital

A Journal of

The Medical Sciences and Hospital Administration.

New Series. No. 191, Vol. VIII. [No. 1263, Vol. XLIX.] SATURDAY, OCTOBER 8, 1910.

### THE SPREAD OF POLIOMYELITIS IN AMERICA.

The severe epidemics of anterior poliomyelitis which have visited New York and other parts of the United States have stirred up much research, both as to prevention and to treatment of the developed disease. That these problems are by no means mastered, or even in the way of being mastered, is shown by the growing uneasiness of the medical profession across the Atlantic. From the latest accounts it would seem that the ravaged zone is tending to widen its borders rather than the reverse. Thus numerous cases are now being reported from districts further south than the original epidemic. Washington has about two hundred recent cases, and fears are entertained that the infection—if it is an infection—may travel further south still. But disquieting though these facts are, it is satisfactory to know that doctors are not content merely to attend the sufferers as they are attacked. In the district of Columbia, for instance, wherein the City of Washington is situated, the local medical association has appointed a committee to investigate and report on the status of epidemic anterior poliomyelitis with respect to ætiology, epidemiology, course, diagnosis, treatment, etc. In the name of the Association the committee has issued an urgent request for support and assistance to all physicians practising in the district who have seen cases of infantile paralysis in private or hospital practice, and a series of questions has been framed, from the answers to which the committee hopes to obtain more light on this serious scourge. All physicians are assured that information furnished will be regarded as professionally confidential; that the data shall not go beyond the custody of the committee; and that the report of the committee will include no personalities, but only the statistical results of the entire investigation. Further, the physician in charge of each case will be the sole agent in supplying the information sought, and no other agency will be employed except on his request or with his approval. With their personal interests thus fully safeguarded, it remains only for the practitioners to co-operate with the committee to the best of their abilty, and if that is fully done it seems quite possible that substantial progress may result.

The points to which the committee is devoting especial attention are mainly as follows: Whether the secretions of the nose and throat are capable of carrying the disease, and if so, when the period of infectivity begins and when it ends. Whether it is possible by any method to recognise the disease with certainty before the onset of paralysis; and if so, what the method is. Whether it is possible by any method to identify abortive or atypical cases and carriers—if carriers exist—who present no typical symptoms. Whether any intermediate host, such as insects or domestic animals, may be responsible for the transmission of the disease. To these ends arrangements have been made in several laboratories to make such investigations as may be necessary. Physicians who have under care patients suffering from acute anterior poliomyelitis, or those suspected of being possibly abortive or atypical cases are invited to communicate with the secretary of the committee, who will, if circumstances warrant it, put him in communication with one of the specialist laboratory workers who have agreed to collaborate. Whenever patients are thus to be made the subjects of study, nothing will be done without the full approval of the physician in charge, nor in any circumstances will anything be permitted which might in any way jeopardise the health of the patient. It is difficult to see anything in the scheme of co-operative research thus outlined which can offend the most delicate susceptibilities, either of medical men or of patients and their friends.

It may be recalled that interesting and valuable results have already been obtained in New York by a joint committee of the Neurological and Pediatrical Societies. It was concluded that the disease is infective but not contagious; that it is more fatal to life than is commonly supposed; but that on the other hand complete recovery without paralysis is also less unusual than was previously thought. It was also found that the lesions, though most marked in the anterior horns, are not confined to them, and therefore the term "anterior" in the nomenclature of the disease is misleading. Not only may all parts of the grey matter show inflammatory changes, but even the white matter also, though to a relatively minor degree.

There were many other important points in the pathology of the disease worked out during the research. For instance, it proved impossible to decide from a study of the pathological histology of the disease whether the infection has a hæmatogenous or a lymphogenous origin. However, while the central nervous system is the seat of the principal lesions in acute poliomyelitis, there are to be found in some of the internal organs of the body changes which point to the probability of a general infection. In particular the acute inflammation of the lymphatic tract of the intestinal system may indicate the path of entrance of the infective agent. One authority (Shaffer) has recently suggested that the avenue of infection is by abrasions of the skin. During the New York epidemic of 1909, Flexner and others of the staff of the Rockefeller Institute made more headway than they had succeeded in doing in the two preceding years. They produced the disease in monkeys repeatedly by the use of the spinal cord of fatal cases, though the fluid obtained during life by lumbar puncture has always failed to be infective. The blood, spleen, liver, and bone marrow are also virulent, but to a very low degree. The virus has not been identified; it is not arrested by the finest filter. Drying for seven days over caustic potash does not diminish its virulence, nor does admixture with glycerine; it even resists five per cent. phenol. But heat readily destroys it; a temperature of 45° C. is sufficient. These facts sufficiently attest that patient research is steadily though slowly approaching to an identification of the *materies morbi*. The disease is so serious and its distribution so universal that the profession everywhere will be only too grateful to the American investigators if their superior facilities (owing to large epidemics) result in enhanced control of this infection.

